# Abstracts and Selections

**Published:** 1905-11

**Authors:** 


### Abstracts and Selections.

While we are ready to indorse the attempts that are being made to place the practice of medicine upon a higher basis, yet we are not led to believe that the great army of medical men throughout our country will marshal themselves at the bugle call of the editor of the Octopus or of *The California State Journal of Medicine* and others. There is a marked degree of independence in the profession that will forever and eternally prevent any attempts to curtail independent action on the part of the individual physician. With the years of hard study of the causation and symptomology of disease, it does not stand to reason that the average physician will habitually prescribe preparations, the composition of which he is in absolute ignorance, and concerning the manufacture of which he is equally ignorant. The statement that the average physician today is as capable of compounding a tonic that will prove both as efficient and palatable as that furnished through our proprietary houses is nonsensical.

Today these subjects are in the hands of those who have given to them a life study, as a result of the demands of the world's progress. Pharmaceutical chemistry today owes its advanced strides to the painstaking efforts on the part of our pharmaceutical houses to supply the physician with both a palatable and efficient preparation.

The question of secrecy or non-secrecy lies, as a matter of fact, with the individual physician. There are at present relatively few preparations, the essential constituents of which would not be divulged by the makers upon application. That they are not divulged is more a result of financial protection against unscrupulous druggists than for the purpose of defrauding the physician. Pharmaceutical preparations differ in no wise from others of the world's goods, their true virtue being in the intrinsic value. The writer can not conceive of any physician prescribing preparations, the composition of which he is in absolute ignorance, and at the same time is of no therapeutical value.

We believe that the great proprietary scare is now over, and that precious few are those men who have been led to modify their system of therapeutics by the appearance of this proprietary ghost. *Texas Courier-Record of Medicine.*

[Almost without an exception, physicians everywhere use certain well known and tried advertised preparations, and it were folly to deny them the right to exercise their own judgment in the matter. It may safely be left to the individual physician to determine for himself the "ethics" of his practice.—Ed.]

---

We must say we have not been able to satisfy ourselves that the American Medical Association has wrought any startling reforms. The fakir, if anything, is multiplying and is especially numerous in the cities of States that have been for some time under the reorganized *regime;* what check they have had has come from local State initiative. What has been accomplished in reciprocity of licensure has been State initiative. What has been done in elevating educational standards seems to have come from State initiative, greater demands of State Boards of Examination, necessarily followed by reforms in curricula. And finally, as regards the weight exerted by a united profession, we would point to the absolute ignoring shown the A. M. A. support of the Pure Food Bill, the failure of the Asociation's own petition for incorporation, the scant attention paid to the Asociation's suggestions of a National Board of Health, a Medical Cabinet Officer, etc.

Have the affairs of the Association been always conducted with the equity and dignity that most certainly should characterize a representative body of the noblest of professions? Frankly, we think not. The trustees have not, to our mind, always shown openness, frankness or fairness. We can not enter now into details, but that there has been "railroading," invidious motions "to table," checking of free debate, etc., no fair or intelligent man can doubt.

And then the *Journal!* Created to be a dignified vehicle of utterance of representative American physicians, and created wholly and solely for that, it has become a gigantic money-scheme—not a white elephant surely, but a whited sepulchre, earning over $40,000 a year. And how? By printing, in the face of repeated protest, advertising matter renegaded in the estimation of the men to whose efforts and interest it owed its birth and continuance. Of late months there is some improvement, the direct result, to our mind, of continuous sarcastic comment from the *California State Journal.* But we all remember when the *Journal* carried that especially delectable "Before" and "After" plate, depicting a rakish, physically-battered female, with the sunken nose, converted in the "After" tableau into a classic Greek by the deft paraffin prosthesis of "Prof." Karl Somebody or Other, of Chicago.—*The Georgian Practitioner.*

---

The International Tuberculosis Congress was opened in Paris on October 2, in the presence of President Loubet, the cabinet ministers, and the ambassadors. Over 3500 delegates were present. The American official delegation consisted of Dr. Henry Barton Jacobs, of Baltimore; Dr. S. A. Knopf, of New York; Dr. Lawrence F. Flick, of Philadelphia, and Medical Inspector Henry G. Boyer, of the United States Navy. Many other Americans, delegates from various societies, were present. The opening address was delivered by Dr. Héard of the French Academy of Medicine, who was chosen president of the Congress. The report of Dr. Letulle, the secretary, said that thirty-five nations were represented in the Congress. An invitation has been extended through the American ambassador to hold the next Congress in this country.

